# The Use of Digitalis in Valvular Disease of the Heart

**Published:** 1907-05

**Authors:** 


### THE USE OF DIGITALIS IN VALVULAR DISEASE OF THE HEART.

The uses of digitalis in valvular cardiac disease are summarized by E. H. Colbeck. He declares that digitalis should seldom, if ever, be given in case of aortic regurgitation which have developed during or after middle life, since under these circumstances the ventricular wall is seldom sound. It should never be given in cases presenting evidence of myocardial degeneration or disease. If complete rest is obtained the drug is permissible, and even beneficial to a certain point in young and otherwise healthy adults who show signs of circulatory failure, more especially when aortic lesion is combined with mitral incompetence. The drug should be discontinued for some time before exercise is resumed. Neglect of this precaution is often followed by rapid ventricular failure and sudden death. In aortic stenosis the drug is contra-indicated, as in this case the augmentation of the tonus of the heart and peripheral vessels (caused by the remedy) would merely increase the obstacle against which the left ventricle has to contend. In mitral incompetency the benefit of the remedy is universally admitted. On the left side of the heart, a rise of cardiac tonus resists the overfilling of the ventricle, which is the initial effort of the valvular insufficiency, while subsequent hypertrophy maintains the tonus of the ventricle and promotes the expulsion of its increased content. On the right side of the heart, a rise of tonus and consequently hypertrophy enable the right ventricle to cope with the increased resistance in the pulmonic circuit, and, by maintaining the blood pressure in the pulmonary veins and left auricle, it resists the reflux through the mitral opening and insures an adequate supply of blood to the left side of the heart. In uncomplicated cases of mitral stenosis the supply of blood to the systemic circulation tends to become increasingly restricted. A rise in the tonus of the left ventricle and peripheral vessels would still further diminish the charge of blood delivered to the aorta. No doubt the effect of increased cardiac tonus on the right side of the heart would be of benefit, provided the blood supply to the left ventricle could be thereby augmented. It may be inferred, therefore, that digitalis can be of no benefit to mitral stenosis in the absence of failure of the right ventricle, and in this event, so soon as the pulmonary blood pressure has been raised to the left ventricle, the drug would again act prejudicially.—British Med. Journal, Dec. 1st, 1906.

